# Eosinophilic Airway Diseases: From Pathophysiological Mechanisms to Clinical Practice

**DOI:** 10.3390/ijms24087254

**Published:** 2023-04-14

**Authors:** Mauro Mormile, Ilaria Mormile, Salvatore Fuschillo, Francesca Wanda Rossi, Laura Lamagna, Pasquale Ambrosino, Amato de Paulis, Mauro Maniscalco

**Affiliations:** 1 Department of Clinical Medicine and Surgery, University of Naples Federico II, 80131 Naples, Italy; 2 Department of Translational Medical Sciences, University of Naples Federico II, 80131 Naples, Italy; 3 Istituti Clinici Scientifici Maugeri IRCCS, Pulmonary Rehabilitation Unit of Telese Terme Institute, 82037 Telese Terme, Italy; 4 Istituti Clinici Scientifici Maugeri IRCCS, Directorate of Telese Terme Institute, 82037 Telese Terme, Italy

**Keywords:** asthma, biomarkers, chronic obstructive pulmonary disease, chronic rhinosinusitis with nasal polyps, eosinophils, disability, rehabilitation, outcome

## Abstract

Eosinophils play a key role in airway inflammation in many diseases, such as allergic and non-allergic asthma, chronic rhinosinusitis with nasal polyps, and chronic obstructive pulmonary disease. In these chronic disabling conditions, eosinophils contribute to tissue damage, repair, remodeling, and disease persistence through the production a variety of mediators. With the introduction of biological drugs for the treatment of these respiratory diseases, the classification of patients based on clinical characteristics (phenotype) and pathobiological mechanisms (endotype) has become mandatory. This need is particularly evident in severe asthma, where, despite the great scientific efforts to understand the immunological pathways underlying clinical phenotypes, the identification of specific biomarkers defining endotypes or predicting pharmacological response remains unsatisfied. In addition, a significant heterogeneity also exists among patients with other airway diseases. In this review, we describe some of the immunological differences in eosinophilic airway inflammation associated with severe asthma and other airway diseases and how these factors might influence the clinical presentation, with the aim of clarifying when eosinophils play a key pathogenic role and, therefore, represent the preferred therapeutic target.

## 1. Introduction

Eosinophils are the key actor in airway inflammation in many conditions such as allergic and non-allergic asthma, chronic rhinosinusitis with nasal polyps (CRSwNP), and in chronic obstructive pulmonary disease (COPD). In these disorders, eosinophils contribute to tissue damage, repair, remodeling, and disease persistence through the production of a variety of mediators, including basic proteins (major basic protein, eosinophil cationic protein, eosinophil peroxidase, and eosinophil-derived neurotoxin), chemokines (CCL5, CCL11, CCL13), cytokines (interleukin (IL)-2, IL-3, IL-4, IL-5, IL-10, IL-12, IL-13, IL-16, IL-25), and growth factors (tumor necrosis factor, transforming growth factor α/β) [1,2]. IL-5 is the key factor responsible for eosinophils accumulation, activation, and terminal differentiation from hematopoietic progenitor cells in both the bone marrow and airways (in situ eosinophilopoiesis), and it is principally secreted by T-helper cell type 2 (Th2) lymphocytes and innate lymphoid cells–type 2 (ILC-2). The knowledge of molecular pathways sustaining the production of IL-5 and, consequently, of eosinophilia in respiratory disease is essential information to choose the most effective biological treatment for each patient. Five monoclonal antibodies (mAb) targeting some molecules participating in type 2 (T2) inflammation (i.e., anti-IL4/13, anti-IL-5, anti-IL-5Rα, anti-IgE, anti-thymic stromal lymphopoietin (TSLP)) are now available for severe asthma and some of them are also indicated for CRSwNP. Moreover, some clinical trials targeting eosinophilic inflammation in COPD are currently ongoing. In order to sharpen the biological drug choice, patient classification based on clinical characteristics (phenotyping) and pathobiological mechanisms (endotyping) has become mandatory [3]. The importance of selecting tailored treatments according to the patients’ features has been deeply investigated by many research groups, especially in severe asthma (SA) [4]. Several phenotypes of SA have been identified according to the age of disease onset, the number of previous exacerbations, the cell types dominating the inflammatory picture, and some comorbidities [5]. According to the expression of type 2 cytokines (IL-4, IL-5, and IL-13), SA has been classified as type 2-high (eosinophilic, T2-high) or type 2-low (non-eosinophilic, T2-low) phenotype [6,7].

T2-high asthma encompasses several asthma subtypes, such as early-onset allergic, late-onset eosinophilic asthma, and aspirin-exacerbated asthma [5]. The secretion of T2 cytokines can be driven by two non-mutually exclusive pathways. The first is sustained by Th2 cells activated by inhaled aeroallergens (allergen-specific IgE) in genetically susceptible individuals. The second is mediated by the ILC-2 upon stimulation with epithelial-secreted alarmins, such as TSLP, IL-25, and IL-33 [8,9]. 

The pathobiology of T2-low asthma is thought to be characterized by neutrophilic inflammation driven by Th1 and/or Th1/17 cell activation [5]. 

Although T2-high asthma is typically associated with eosinophils, their presence does not automatically mean that they play the most relevant pathogenetic role and that their ablation will be accompanied by a clinical response. Similarly, in subjects with systemic eosinophilia the presence of the atopic state does not guarantee an effective response to mAbs designed for patients with atopic asthma (e.g., anti-IgE or anti-IL4R). However, it is equally true that biological treatment can often lead to clinical remission of asthma [10], suggesting that the expected outcome of treatment can be achieved by targeting the specific drivers (endotypes) of the disease.

In this review, we describe some of the immunological differences in eosinophilic airway inflammation associated with SA and other airway diseases and how these factors might influence clinical presentation, with the aim of clarifying when eosinophils play a key pathogenic role and, therefore, represent the preferred therapeutic target. 

## 2. Eosinophilopoiesis

Eosinophil development and maturation largely occur in the bone marrow from CD34^+^ hematopoietic precursors upon stimulation with IL-3, GM-CSF, and IL-5, which bind to receptors sharing a common beta chain. 

IL-5 receptor, formed by a unique binging α chain (IL-5Rα), is upregulated on CD34^+^ cells at an early stage in eosinophil-lineage-committed progenitor cells (EoP) [11]. IL-5 plays a central role in the production, mobilization, activation, recruitment, and survival of eosinophils at the site of inflammation [12]. The expression of IL-5Rα on EoP is increased in the bone marrow of atopic asthmatics suggesting that, in atopic subjects, EoP are primed to promptly respond to IL-5 [13].

In addition to Th2 lymphocytes and ILC-2s, further sources of IL-5 are EoPs, invariant natural killer T cells, and mast cells [14]. However, IL-5 can be released by eosinophils themselves in an auto/paracrine manner [15].

Eosinophils recruitment from the blood into organs and tissues are favored by some chemokines, such as CCL5 (RANTES), CCL7 (MCP3), CCL11 (eotaxin 1), CCL13 (MCP-4), CCL15, CCL24, and CCL26. These chemokines act as eosinophil chemoattractant and bind to the CCR3 chemokine receptor, along with type 2 receptors (DP2 or CRTH2) and its ligand prostaglandin (PG) D2, orchestrating synergistically with IL-5 the migration of eosinophils from blood to the lungs [2,16,17].

There are data supporting the view that local maturational processes sustain the development of eosinophilia in the lung. Indeed, anti-CCR3 treatment has been reported to be insufficient to clear luminal airway eosinophils in moderately SA, likely because the treatment did not attenuate local eosinophil differentiation reflected in the lack of effect of the drug on sputum levels of either EoPs or mature eosinophils [18]. 

Th2 cells favor eosinophil activation and survival by releasing primarily IL-5 [19]. These data support the view that in situ eosinophilopoiesis promotes the development of lung eosinophilia and suggest that targeting local IL-5-driven expansion of EoPs may be an important approach to control persistent airway eosinophilia and clinical response to anti-eosinophil treatments in SA [20,21]. 

## 3. Eosinophils and Bronchial Asthma

### 3.1. Eosinophils in Type 2-High Asthma Phenotype

T2-high asthma phenotype as compared to T2-low phenotype is associated with higher blood eosinophil count, a greater expression of IL-5 in airway mucosa, more severe airway hyper-responsiveness, larger total serum IgE, and higher fractional exhaled nitric oxide (FeNO) [22]. 

IL-5 and IL-4/IL-13 are considered the key drivers of type 2 pathways underlying eosinophilic airway inflammation in asthma. 

In allergic asthma, in genetically predisposed individuals, naïve CD4^+^ T cells are activated via presentation of allergens by dendritic cells differentiating into Th2 cells. Th2 cells produce cytokines, such as IL-4, IL-5, and IL-13, and lead to IgE switching from B cells, airway eosinophilia, and mucous hypersecretion [23]. In eosinophilic non-allergic asthma, the interaction of environmental pollutants, microbes, and glycolipids with airway epithelium induce the release of IL-33, TLSP, and IL-25 [24]. These epithelial-derived alarmins promote, in an antigen-independent manner, a T2-mediated inflammation by acting on ILC-2 cells, which, when activated, secrete high amounts of IL-5 and IL-13 that orchestrate the accumulation, activation, and differentiation of EoP in peripheral tissues [25]. The key pathogenetic role played by eosinophils in bronchial asthma is supported by some observation. Asthma severity correlates with the level of blood and airway eosinophils, and, in addition, treatments reducing sputum eosinophils level are able to reduce asthma exacerbations and spare corticosteroid use in patients with severe asthma [26].

**Figure 1:**
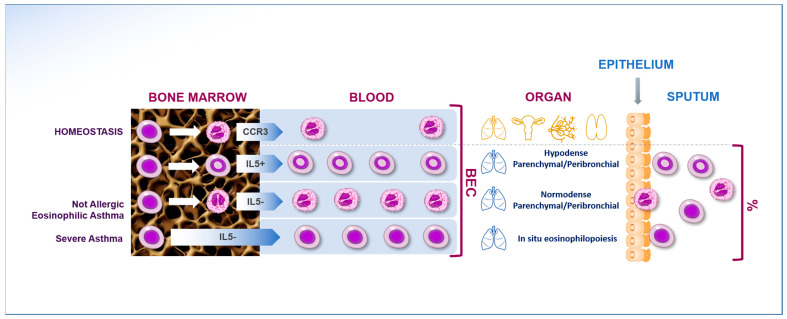
Eosinophil endotypes derived from different signals perceived by the developing progenitors of eosinophils. There may be a different expression of eosinophil subpopulation differing from their localization (parenchymal/peribronchial and in situ), cellular density profiles (hypodense/normodense), asthma endotypes (allergic/non-allergic eosinophilic), and responsiveness to IL-5.

FeNO and blood and sputum eosinophil count have been reported to predict T2-high phenotype in SA [27]. Indeed, these biomarkers are being used to select SA candidates to receive targeted T2 therapies such as anti-IL-5 or anti-IL-5Rα treatment and to anticipate the therapeutic response. Interestingly, in a non-selected population of SA, there was an overall discrepancy in the coexpression of biomarkers related to IL-5 and IL-13 pathways, suggesting the existence of distinct subtypes of T2-high SA [28]. Nonetheless, all the biological drugs available to date for the treatment of T2-high phenotype have shown a better response at the increasing number in blood eosinophil levels. However, considering only the number of circulating eosinophils in order to identify patients with eosinophilic-driven airway inflammation can often be misleading. Indeed, there is evidence for the existence of several eosinophil endotypes derived from different signals perceived by the developing progenitors of eosinophils. Data from mouse models, also confirmed in asthmatic patients, show that among asthma phenotypes there may be a different expression of eosinophil subpopulation differing from their localization (parenchymal/peribronchial), cellular density profiles (hypodense/normodense), asthma endotypes (allergic/non allergic eosinophilic), surface adhesion capacity, and responsiveness to IL-5 [29]. In addition, regulatory and inflammatory subsets of eosinophils have also been documented in a murine model of eosinophilic esophagitis [30,31] (Figure 1).

The ability to distinguish systemic eosinophilia with pathogenetic relevance in the airways from systemic non-inflammatory eosinophilia as an epiphenomenon linked to specific/non-specific stimuli promoting the production of Th2 cytokines helps to define whether eosinophils drive the disease.

### 3.2. Eosinophils in Type 2-Low Asthma Phenotype

In type 2-low asthma, the upregulation of T2 immune pathways (e.g., IL-4 and IL-13) and eosinophilic inflammation characteristic of type 2-high phenotype are classically absent, contributing to the poor response to corticosteroids [32]. Although the pathogenesis of type 2-low asthma is not completely elucidated, the following mechanisms have been suggested to play a role in this subtype of asthma: (i) type 1 (IFN-mediated) and type 3 (IL-17-mediated) immune pathways together with airway neutrophilia; (ii) pauci-granulocytic mechanisms; (iii) systemic inflammation associated with IL-6, obesity, and metabolic dysfunction [32]. In particular, murine models show that IL-17 contributes to neutrophilic airway inflammation, bronchial hyper-responsiveness, mucus hypersecretion, airway obstruction, and corticosteroid resistance [33,34,35]. In addition, levels of Th17-derived IL-17 in the airway and peripheral blood correlate with disease severity [34,36]. Despite this evidence, recent studies demonstrate that patients with severe type 2-low asthma do not respond to anti-IL-17 treatments [37].

Eosinophilic and neutrophilic asthma are not mutually exclusive subtypes. The concomitance of activation of type 2 immune pathways in these patients may sometimes occur, further worsening the clinical and functional outcome. Indeed, in the airway of patients with SA, eosinophils can accumulate together with neutrophilic count increase [38]. In addition, the inflammation pattern may change in the same patient over time. In a large retrospective study [39], the authors analyzed 1786 consecutive sputum cell counts from 1139 patients with airway disease, reporting changes in the cellular nature of airway inflammation among consecutive exacerbations in 48% of the patients. The existence of a dynamic interplay between type 2-high and type 2-low pathways in different phases of airway inflammation is supported also by the identification in the circulation of patients with bronchial asthma of a subset of T cells (Th17/Th2 cells) that can secrete both IL-4 and IL-17 [40]. These findings strengthen the hypothesis that different inflammatory phenotypes can coexist in one patient and alternate according to the different stages of the disease.

### 3.3. Early-Onset Allergic Disease vs. Late-Onset Eosinophilic Airway Disease

Approximately 40–50% of all patients with asthma develop the disease during childhood (early-onset asthma). Generally, these patients are atopic with multiple sensitizations to allergens [41]. High total IgE, high FeNO levels, and increased sputum and blood eosinophils characterize patients with severe early allergic asthma. In these cases, airflow limitation and airway remodeling could appear early.

Late-onset eosinophilic asthma phenotype, often non-allergic, is present in about 25% of SA patients and present persistent airflow limitation, accelerated decline of lung function, often requiring high doses of inhaled corticosteroids (ICS) or being refractory to oral corticosteroids (OCS) treatment [42]. Another recognizable clinical characteristic, especially in severe late-onset asthma, is the concomitance of CRSwNP [43] and blood eosinophils count [44,45]. It has been suggested that ILC-2 activation plays a role in both early-onset allergic asthma and in the late-onset asthma with CRSwNP phenotype [46]. 

It has been widely demonstrated that in the late-onset eosinophilic phenotype, the presence of allergy does not represent a factor impacting the efficacy of the anti-eosinophilic therapeutic approach [47,48], demonstrating how this eosinophil-driven phenotype is untied to allergenic stimuli and therefore could not be effectively modulated using drugs targeting T2 inflammatory cytokines (anti IL-5 and IL-4/13) or anti-IgE. This observation resembles the immunological phenotype switch observed in patients with eosinophilic esophagitis in which children seem to respond more to target food elimination than adults [49]. Discrepancies in response between children and adults may suggest a decreasing pathogenic role of IgE in the transition from childhood to adulthood.

### 3.4. Current and Novel Biomarkers of Eosinophilic Airway Inflammation 

The new therapeutic frontiers in SA reflect the need to identify reliable biomarkers capable of identifying different phenotypes and endotypes of asthmatics to guide the therapeutic choice. Table 1 summarizes the main molecules studied so far for this purpose.

Blood eosinophil count is a commonly used indicator of T2 inflammation in routinary clinical practice. It is easily measured and reproducible. In addition, it has been shown to correlate with risk of exacerbations in patients with SA [68]. Moreover, eosinophils levels ranging from 150 to 400 cell/µL can also predict the response to treatment with mAbs, targeting the IL-5 pathway. The MENSA and DREAM studies pointed out that patients with levels of eosinophils ≥ 150 cell/µL have a better response to mepolizumab (mAb targeting IL-5) [69,70]. Otherwise, in SIROCCO and CALIMA studies, patients with severe asthma and eosinophils ≥ 300 cell/µL had a better response to benralizumab (mAb targeting IL-Rα) [71,72]. Finally, studies conducted with the use of reslizumab (mAb targeting IL-5) showed a reduction in exacerbation rate and improvement in lung function in patients with eosinophils ≥ 400 cells/µL [52]. However, eosinophils as a biomarker have low specificity since they can be elevated in several autoimmune and atopic diseases. In addition, non-respiratory diseases influence B-Eos count but not FeNO or CRP. Male sex, obesity, certain races/ethnicities, and current smoking are individual characteristics or exposures that are associated with higher B-Eos counts [73]. Moreover, a single determination of blood eosinophil count is often inadequate. Indeed, the Global Initiative for Asthma (GINA) guidelines suggest the need for at least three measurements to identify eosinophilic asthma phenotype [74,75]. In addition, based on the International Severe Asthma Registry (ISAR), patients with SA are classified as eosinophilic phenotype, based not only on blood eosinophil count but also on some clinical features (i.e., OCS dependency, presence of CRSwNP, elevated FeNO, and late onset of the disease) [76]. These clinical features are closely associated with the eosinophilic asthma phenotype because their prevalence increase as the number of blood eosinophils increases, thus suggesting that they may be directly linked with the eosinophilic phenotype and be predictor of successful response to anti-eosinophilic treatment [47,48,49,77,78,79]. Another key point is given by the fact that some scientific evidence has shown discordance between blood and sputum eosinophil count, particularly observed in OCS-dependent asthmatics [80]. These findings do not support the role of absolute eosinophil count as the only biomarker for anti-IL-5 mAb treatment response. This discordance is likely due to in situ eosinophilopoiesis, resulting from incomplete neutralization of IL-5 produced in airways by ILC-2 cells. Moreover, in a retrospective study on 508 asthmatics with successful sputum induction, Schleich et al. [81] investigated the prevalence and characteristics of patients with concordant and discordant systemic and bronchial eosinophilia. Asthmatics with isolated systemic eosinophilia (7%) had similar characteristics as non-eosinophilic asthmatics. The group with concordant systemic and airway eosinophilia (19%) showed remarkable male predominance, and had the lowest airway caliber, asthma control, and quality of life, and the highest airway hyper-responsiveness, exacerbation rate, and FeNO. 

FeNO is an indirect biomarker of airway type 2 inflammation, and its production is enhanced by the IL-13 and IL-4 pathways that promote the activity of NO synthase [82,83,84,85]. Eosinophilic inflammation is highly probable with FeNO levels above 50 ppb and unlikely when FeNO levels are below 25 ppb [86]. From a clinical perspective, FeNO has a potential role in guiding adjustments in dosing ICS, with a reduction in exacerbation rate [86]. Furthermore, FeNO levels are higher in naïve corticosteroids patients, so it can be used as an indicator of poor patient compliance. 

FeNO levels also correlate with the therapeutic response to dupilumab (anti-interleukin-4 receptor α mAbs that blocks both interleukin-4 and interleukin-13 signaling) since several studies showed that FeNO levels decreased during treatment with this drug [87,88]. Contrarily, FeNO is not affected by biologics targeting IL-5 as mepolizumab. Indeed, mepolizumab has been shown to decrease consistently the blood and sputum eosinophil count in patients with eosinophilic asthma, with no effect on FeNO levels [89]. FeNO is considered a biomarker of T2-driven airway inflammation. Although it correlates with airway eosinophilia in steroid-naïve patients, it can be independent from eosinophils and therefore not helpful in monitoring the response to anti-IL-5 mAb [90]. However, the prognostic and predictive value of FeNO as a biomarker may have several limits, as it can be influenced by several confounders, including age, height, sex, and recent respiratory infections [70]. In addition, FeNO is strongly associated with atopy and may be increased in the absence of eosinophilic inflammation [91,92]. 

Current and previous evidence have suggested FeNO as a surrogate marker for sputum eosinophilia [93]. Contrariwise, low FeNO despite high sputum eosinophils has been reported in some adult asthmatic patients [94]. Other evidence about the decoupling of FeNO from sputum eosinophilia came from a study by Wenzel et al., in which no improvement in the levels of blood or sputum eosinophilia was found with dupilumab despite the decrease in other biomarkers such as FeNO and the improvement of forced expiratory volume in 1 s (FEV_1_) [25]. In the study by Crespo et al. [12], a discrepancy between FeNO and sputum eosinophil count was observed in about 42% of patients. Of these patients, 73.9% with a predominance of non-allergic asthma had a FeNO <50 ppb and high eosinophil count. The remaining patients with a predominance of atopy and pauci-granulocytic inflammatory phenotype (26.1%) had FeNO ≥50 ppb and normal sputum eosinophilic count.

The causes of these observations are not completely elucidated. A possible explanation may be found in the differences between molecular pathways leading to increase in FeNO and recruitment and activation of eosinophils. Overall, given the above conflicting evidence, further ad hoc studies are needed to clarify whether FeNO could be effectively considered in the near future for routine use as a biomarker of airway eosinophilia in different clinical and healthcare settings (i.e., community, hospital, rehabilitation) [95,96].

Another molecule that has been proposed as surrogate marker of Th2 inflammation in SA is periostin, an extracellular matrix proteinase, produced by bronchial epithelial cells in response to IL-4 and IL-13. Periostin is also implied in asthma airway remodeling, as it promotes activation of fibroblasts and has autocrine effects on the epithelial cells [57]. As a biomarker, higher levels of periostin correlate with higher exacerbation rate and poor asthma control [97]. Serum periostin levels respond partially to ICS therapy, possibly reflecting a reduction in airway inflammation and wall thickening in asthma [98]. In addition, biologics targeting IL-4/1L-13 (dupilumab) [99,100] and IL-13 (i.e., lebrikizumab) [101,102] have been shown to suppress serum periostin levels in asthma patients.

However, periostin has low specificity, since its levels are influenced by age and other disease as atopic dermatitis, scleroderma, cancer, and diabetic nephropathy [74].

T2-high asthma phenotype hosts up to 70% of SA patients and occurs in both allergic and non-allergic asthma. Owing to difference in blood eosinophil count [103], age at disease onset (early vs. late), clinical presentations (e.g., influence of allergy on symptoms), and presence of comorbidities (e.g., CRSwNP, atopic dermatitis, and obesity), T2-high asthma phenotype represents a highly heterogeneous group [43]. Thus, the conventional available biomarkers of T2 pathways (blood eosinophil count, FeNO, and serum IgE) are both individually or in combination unpowered to identify and rank the driver of the disease (e.g., IL-4, IL-5, IL-13, or IgE) [27]. The picture is further complicated because these biomarkers are not necessarily correlated with each other. In a study by Frossing et al. [28], the elevation in at least one biomarker was reported in 70% of patients with SA, while the simultaneous increase in all three biomarkers was observed in only 15%. The prevalence of elevation of each biomarker in isolation was reported in about 40% of the study group. However, the combined use of FeNO and blood eosinophils count should be recommended as they offer independent information in relation to risk for asthma morbidity [104].

This observation suggests that the degree of activation of different T2 pathways varies among patients, underscoring the importance of multiple domains characterization of T2 pathways in order to achieve better patient stratification. 

## 4. Eosinophilic Airway Inflammation and Comorbidities

Upper and lower airways are considered a unified morphological and functional unit, and the connection existing between them has been observed for many years [105]. About 60% of patients with CRSwNP present comorbid asthma (with and without NSAID-exacerbated respiratory disease), predominantly atopic and not severe [106]. Accordingly, about 25% of patients with severe eosinophilic asthma have a comorbid CRSwNP [77]. 

Increased nasal polyposis and oral steroid courses for chronic rhinosinusitis (CRS) are reported in late-onset severe asthma as compared to early-onset asthma [107]. In CRS, as in asthma, the activation of ILC-2 cells leads to the release of cytokines (Il-9, Il-4, Il-5, and IL-13) stimulating a Th-2 inflammatory response [108,109]. As for asthma, there are different inflammatory phenotypes and endotypes of CRS. Diverse immune cells and inflammatory mediators orchestrate this heterogeneous disease spectrum, which comprises CRSwNP and CRS without nasal polyps (CsNP) [110,111,112]. Two distinct phenotypes of CRSwNP have been described, one with eosinophilic CRS (ECRSwNP) and one with non-eosinophilic CRS (NECRSwNP). ECRSwNP is a disabling condition associated with severe eosinophilic infiltration and more severe sinus inflammation on sinus CT scan and nasal endoscopy compared to those with NECRSwNP [113]. Tissue eosinophilia was also a predictor of nasal polyp recurrence following surgery, another indicator of more recalcitrant disease. The diagnosis of ECRS is made by examination of a biopsy specimen or resected tissue [114]. Moreover, for determining the severity of ECRS several factors have been proposed, including peripheral blood eosinophil, ethmoid sinus-predominant involvement on CT scans, and the presence of complications (current asthma or a history of asthma, aspirin intolerance, and/or NSAID allergy). On the other side, blood and sputum eosinophil levels in patients with asthma are directly correlated with sinus mucosal thickening osteitis and are associated with increasing number of prior sinus surgery, lending further support to the hypothesis that similar inflammatory processes [115] mediate both asthma and CRS.

There is still no standardized process to establish the most appropriate therapy for asthmatic patients with nasal polyps. What is emerging quite clear from both randomized and real-world evidence studies is that, more than an asthma comorbidity, the presence of nasal polyposis is a hallmark of late-onset eosinophilic asthma identifying a phenotype particularly responsive to anti-eosinophilic therapy with improvement of quality of life, lung function, and reduction in asthma exacerbations [116]. The presence of CRSwNP in patients with SA is accompanied by a more extensive eosinophilic inflammation and therefore it can benefit more from a direct anti-eosinophilic approach [117]. 

Anti-IL-5 and anti-IL-5Rα in real-life studies have shown to significantly reduce eosinophils number in nasal polyps, the latter with a complete reduction in eosinophils- and neutrophils-infiltrated cells, with a significant reduction in the nasal polyp score (NPS) after 6 months [118]. However, despite a significant improvement in nasal symptomatology, as assessed by the Sinonasal Outcome Test 22 (SNOT-22) questionnaire, achieved by these anti-eosinophilic drugs, as for other biologics, not all patients showed significant clinical improvement based on computed tomography (CT) and endoscopic results. The variability in the therapeutic response observed in these patients is linked to the great heterogeneity of the disease and further investigations aimed at better characterizing and phenotyping of CRSwNP are necessary.

Another comorbidity which should be actively investigated and treated as a possible trigger factor of uncontrolled asthma is gastroesophageal reflux disease (GERD) [119,120]. This condition should also be ruled out before SA is diagnosed and biologic therapy is started. Both eosinophilic type 2 inflammatory changes and neutrophilic inflammation have been described in patients with GERD [120,121].

Obesity has been associated to airway inflammation and is generally considered a feature of late-onset non-eosinophilic asthma [122]. However, some studies have reported higher levels of airway IL-5 and eosinophils in obese patients in general [123], and the Severe Asthma Research Program (SARP) has also identified a group of predominantly females with severe late-onset asthma, obesity, and increased sputum eosinophils [124]. Therefore, obesity may be associated to both eosinophilic and neutrophilic asthma.

The role of eosinophils is under investigation in several other chronic respiratory conditions including allergic fungal airway disease and chronic idiopathic eosinophilic pneumonia [1,125,126,127].

Systemic hypereosinophilic diseases including eosinophilic granulomatosis with polyangiitis (EGPA) can also be associated with airway inflammation and severe asthma [119,128,129]. An early identification of these disabling diseases is essential as they require a multidisciplinary approach and systemic corticosteroids and/or immune-suppressant therapies.

## 5. Eosinophils and Chronic Obstructive Pulmonary Disease

In recent years, understanding of the pathogenetic mechanisms involved in COPD has greatly increased [130]. Indeed, the awareness of the wide range of inflammatory patterns that can be observed in this disabling condition led to the identification of more personalized rehabilitation and pharmacological approaches [131,132,133,134,135], with a potential role for biologically targeted therapies. The inflammatory pattern in COPD is extremely variegated. In most of the cases, the inflammatory environment in the airways of COPD patients is dominated by neutrophils, cytotoxic CD8+ T cells, and alveolar macrophages [136]. However, eosinophils may also play a key role in a subset of COPD patients [2,137]. 

In fact, several studies have shown that eosinophils may be involved in the inflammatory response in COPD, as under certain circumstances, inflammatory cues promote eosinophil recruitment to the lungs, where secretion of a variety of chemokines and cytotoxic granular products contributes to inflammation [138]. 

Increased levels of eosinophils can be detected in the sputum of approximately one-third of COPD patients, overcoming the old-fashioned concept that eosinophilia is a feature that can be used for distinguishing COPD from asthmatic patients [139,140]. In addition, sputum eosinophilia is particularly increased during COPD exacerbations [140]. Additionally noteworthy is that blood and/or sputum eosinophilia is a biomarker of beneficial responses to inhaled or systemic glucocorticoids for preventing or treating the exacerbations, respectively [2,141]. Evidence from the Evaluation of COPD Longitudinally to Identify Predictive Surrogate End-Points (ECLIPSE) study have shed light on the prevalence of eosinophilic inflammation in COPD patients, pointing out that the elevation of eosinophils in COPD patients is intermittent rather than constant [142]. Indeed, 37.4% of patients with COPD had blood eosinophil counts persistently elevated (≥2%) over a three-year follow-up period, whereas 49% had intermittent elevation of these cells. In addition, the authors described some common features among COPD patients with eosinophil counts persistently ≥2% (i.e., older age, male gender, fewer current smokers, higher FEV_1_% predicted, higher fat-free mass index, fewer symptoms, and lower BODE (body mass index, airflow obstruction, dyspnoea, exercise capacity index) [142]. Interestingly, in the ECLIPSE study, although the subjects with high blood eosinophil counts had better clinical characteristics at baseline, they also showed increased rate of emphysema progression, further confirming the pathogenetic role of this cell in airway inflammation, tissue damage, and remodeling [142,143]. In addition, exploring the relationship between lung function and sputum eosinophilia, several studies have shown a link between airway obstruction and eosinophilic lung inflammation. Indeed, a higher concentration of both eosinophils and their products, such as eosinophil cationic protein, have been related to worse lung function, as expressed by lower FEV_1_ values [144].

The above reported findings about the persistence of eosinophilia in COPD patients are also in contrast with data from the Acute Exacerbation and Respiratory Infections in COPD (AERIS) study, which reported persistent blood eosinophilia ≥2% in most of their cohort (58%) over 1 year [145]. 

A few studies have attempted to establish a relationship between eosinophils blood count and the development of pneumonia in COPD patients, considering also the antimicrobial defense role of these cells, with mixed results. Some authors report no difference in pneumonia incidence between COPD patients with low and high eosinophils levels [146], while other research groups found that patients with higher eosinophils levels (≥2%) had slightly fewer episodes of pneumonia [141,147].

In conclusion, further studies are needed to better elucidate the fluctuation of eosinophils levels in COPD patients and correlate them with lung function and clinical outcome in long-term follow-up.

## 6. Target Therapies in Eosinophilic Airway Disease

### 6.1. Eosinophils and Oral Corticosteroid Dependence

OCS dependence is reported in a subset of SA phenotype that, although representing only a small proportion of the asthmatic population, requires personalized treatment because of its increased morbidity, hospitalization, and mortality. 

These patients remain symptomatic and present persistent airway eosinophilia despite treatment with high-dose ICS and OCS. Several cellular and molecular mechanisms related to immunological dysregulation, genetic and environmental factors, or respiratory infections have been related to OCS insensitivity in asthma [148]. 

Eosinophils, during their migration from the bone marrow to tissues and then to airway lumen, undergo phenotypic changes in response to tissue microenvironmental cues, including altered morphology, enhanced responsiveness to cytokines, different susceptibility to chemotactic mediators, and various degrees of cellular activation, likely acquiring a different susceptibility to pharmacological treatments [149,150,151]. So, indirect anti-eosinophil approaches may not be completely effective in reducing eosinophil number at all degrees of asthma severity, in all compartments and/or for all T2-high phenotypes [152]. In a recent real-life clinical study, suboptimal responses were observed in 42.8% of 250 moderate-to-severe asthmatic patients treated with anti-IL-5 mAbs (i.e., mepolizumab, reslizumab). In that study, daily prednisone requirement, sinus disease, and late-onset asthma diagnoses were the strongest predictors of suboptimal response. Importantly, after 4 months of treatment, reduced blood and sputum eosinophil values were available in 129 patients: 65 patients were suboptimal responders and in 78% of them sputum eosinophil value was >3% [153]. These data, on the one hand confirm the strong correlation between sputum eosinophilia and OCS dependence, and on the other hand, are in line with the observation of the incomplete reduction in eosinophils in blood and tissues in IL-5 or IL5-Rα knockout mouse model, suggesting that IL-5 may not be the only driver of eosinophilia [154]. 

Tissue eosinophils may be resilient to some extent against anti-IL-5 therapies because molecules other than IL-5, such as IL-3 and GM-CSF, can sustain them. The latter was also identified as a predictor of suboptimal response for anti-IL-5 mAbs treatment [153]. 

These data are in contrast with what has been observed in 18 patients with SA and elevated blood eosinophils who relied on OCS therapy to control their disease. In this subset of patients, anti-IL-Rα (benralizumab) showed a significant reduction in both circulating and sputum numbers of mature eosinophils and EoP cells, suggesting that targeting IL-5Rα+ cells decreases EoP cell numbers both systemically and within the airways, thereby attenuating the potential IL-5–driven in situ eosinophilopoietic processes that may contribute to persistent airway eosinophilia in patients with severe, prednisone-dependent asthma [155]. Collectively, these data support the substantial difference existing between a direct and indirect approach to eosinophils.

### 6.2. Biological Therapies and Eosinophilic Inflammation in Severe Asthma

In recent years, a wide range of mAbs targeting IgE or type 2 cytokines have been proved to be highly effective and safe in reducing symptoms and exacerbation in patients with severe allergic and eosinophilic asthma [156]. However, these therapies are not suitable for about half of patients with severe asthma who often present with non-allergic, non-eosinophilic type 2-low asthma [60,157,158]. For this reason, in the era of precision medicine, investigating the different pathways involved in the different endotypes of asthma can help identify new targeted therapies (Table 2). 

In SA, the identification of specific biomarkers has become the basis for a personalized approach that has replaced the conventional treatment based on corticosteroids and bronchodilators drugs. However, the response to biologics targeting T2 pathways is variable and often unpredictable. Although the origin of this variability is multifactorial, the differences in baseline patient characteristics and the complexity and heterogeneity of biological pathways orchestrating T2 responses are the major determinants. The role played by individual cytokines in T2 inflammatory processes is variable as each of them may be involved to a greater or lesser extent in the initiation, maintenance, and amplification of the inflammatory process. A further complication derives from the fact that in the individual patient, the cytokines and the cells on which they act can vary during the course of the disease and in the different target organs. As a consequence, targeting a single cytokine generally does not result in complete abrogation of the inflammatory response in a large majority of patients. 

The selection of the most appropriate biologic for each patient should be made on the basis of the patient’s clinical characteristics (atopy, blood eosinophil count, sputum eosinophil count, and FeNO level) and taking into account the patient’s comorbidities (CRS, atopic dermatitis, urticaria, and obesity) [168].

Patients with asthma that is clearly driven by a clinical history of allergies (rather than just an elevated IgE level) are candidates for anti-IgE therapy.

In subjects with severe T2 eosinophilic phenotype asthma, the presence of comorbidities affecting the upper airways increases as peripheral eosinophilia increases and, therefore, patients with these characteristics may benefit more from treatment with IL-5/Rα, even if the anti-IL-4/13 and anti-IgE treatment are recommended as first-line treatments in these patients. However, in asthmatic patients with overlapping phenotypes, the choice of the biological drug able to guarantee the best therapeutic response is still an unsolved problem.

### 6.3. Biological Therapies and Eosinophilic Inflammation in CRSwNP

Several mAb targeting T2 inflammation (IL-4Rα, IgE, and IL5/Rα) have been approved or are currently in phase 3 clinical trial as add-on therapy for CRSwNP. Dupilumab was the first Food and Drug Administration (FDA)-approved biologic agent in the United States and Europe for the treatment of CRSwNP. Two wide, double-blind, placebo-controlled, international phase 3 studies (LIBERTY NP SINUS-24 and LIBERTY NP SINUS-52) enrolling 724 patients with refractory CRSwNP, assessed the efficacy and safety of dupilumab (300 mg every 2 weeks or 4 weeks subcutaneously) in improving nasal symptoms, disease specific quality of life, use of systemic corticosteroids, and/or the need for nasal polyp surgery [106]. 

Some of the strongest evidence regarding the effectiveness of mepolizumab in improving nasal polyp size and nasal obstruction in CRSwNP, which have led to the approval of the drug for this disabling condition, came from the SYNAPSE study [169].

In this randomized, double-blind, placebo-controlled, parallel group study, the effect of subcutaneous mepolizumab was evaluated in 407 adult patients with highly symptomatic CRSwNP uncontrolled by previous surgery and treated with intranasal steroids. The treatment demonstrated significant improvement in terms of size of nasal polyps and nasal obstruction at week 52 compared with placebo.

In a study conducted in 44 subjects with severe eosinophilic asthma and CRSwNP, mepolizumab administered for one year achieved clinical improvement in both asthma and rhinosinusitis. In addition, the mean percent of eosinophil count in nasal cytology, as compared to baseline, significantly decreased after 6 and 12 months of treatment, suggesting that clinical efficacy of mepolizumab is related to reduced inflammation at tissue level [170]. Similar results have been reported by Walter et al. [171].

Omalizumab has also shown significant superiority to placebo in the POLYP I (*n* = 138) and POLYP II (*n* = 127) phase 3 trials conducted over a treatment period of 24 weeks followed by a 4-week follow-up period [159], and it is currently indicated as an add-on therapy with intranasal corticosteroids when this treatment does not provide adequate disease control. 

Benralizumab has shown promising results in the phase 3 OSTRO study, including 413 randomized patients with severe CRSwNP (207 in the benralizumab group and 206 in the placebo group), demonstrating a reduction in nasal polyps score and improved nasal symptoms as nasal obstruction and difficulty with sense of smell [172]. Another phase 3 trial evaluating efficacy and safety of benralizumab in ECRSwNP (ORCHID; NCT04157335) is currently ongoing. A prospective observational study (NCT03369574) aimed at monitoring CRS symptoms in asthma patients undergoing treatment with reslizumab, evaluating the potential benefit on CRS symptoms, has been withdrawn.

No documented study in CRSwNP is presently available for mAb targeting IL-13, tralokinumab, and lebrikizumab.

### 6.4. Biological Therapies and Eosinophilic Inflammation in Chronic Obstructive Pulmonary Disease

Although there is evidence supporting the existence of eosinophilic inflammation in a subgroup of COPD patients, findings from phase 2 and 3 trials evaluating the efficacy of T2-targeted therapies in COPD have been disappointing as compared to those in asthma patients [137].

A pilot randomized clinical trial (NCT01463644) evaluating 18 subjects treated with mepolizumab given once a month intravenously at the dose of 750 mg for 6 months versus placebo, demonstrated a reduction in blood and sputum eosinophils, without any significant change in FEV_1_, diffusing capacity of the lung for carbon monoxide values, radiological evidence of remodeling, COPD Assessment Test, Chronic Respiratory Disease Questionnaire, and exacerbation rate [173]. Other phase 3, randomized, double-blind, parallel-group trials, namely METREX (NCT02105961) and METREO (NCT02105948), compared the efficacy of subcutaneous injection of mepolizumab given, respectively, at the dose of 100 or 300 mg every 4 weeks in patients with eosinophilic-phenotype COPD patients with history of moderate or severe exacerbations [174]. The authors reported a lower annual rate of moderate or severe exacerbations than placebo among patients with higher blood eosinophil counts at screening, with no differences in patient-reported outcomes and/or lung-function end points [174].

A randomized, double-blind, placebo-controlled, phase 2a study investigated efficacy and safety of benralizumab subcutaneously, every 4 weeks (three doses), then every 8 weeks (five doses) over 48 weeks in a cohort of 101 patients with moderate-to-severe COPD and a sputum eosinophil count of 3·0% or more within the previous year, reporting no significant reduction in the rate of acute exacerbations [175]. Similarly, the TERRANOVA (NCT02155660) and GALATHEA (NCT02138916) randomized placebo-controlled studies analyzed the effect of benralizumab (30 or 100 mg in GALATHEA; 10, 30, or 100 mg in TERRANOVA every 4 weeks for the first three doses and then every 8 weeks) versus placebo on the annualized COPD exacerbation rate in patients with blood eosinophil counts ≤220/mm^3^ gave disappointing results [176].

Other biological drugs investigated in moderate-severe COPD patients with T2 inflammation are dupilumab, for which two clinical trials are currently ongoing (NCT04456673; NCT03930732) and lebrikizumab (VALETA, NCT02546700), which have been completed, but the results are not yet available. The details of these studies are available at the website www.clincaltrials.gov. No documented study in COPD is currently available for reslizumab and tralokinumab.

Collectively, this evidence demonstrates that the role of eosinophils in COPD is more complex than in asthma. Therefore, in order to obtain a substantial improvement in patients’ outcome and respiratory function tests, it would be necessary to act on multiple pathways or cells other than eosinophils, such as T and B cells, macrophages, basophils, and mast cells. 

## 7. Conclusions

In recent years, understanding the role of eosinophil as the driver of pathology in respiratory diseases has increased. The role of eosinophils as biomarkers of T2 inflammation is now established. Furthermore, new eosinophil-targeting therapies have been developed, allowing for better management and therapeutic care of patients. However, the full immunological differences across the spectrum of T2 eosinophilic inflammation and how these differences might influence the clinical presentation and evolution of the diseases are not completely known. Further studies should help understand the contribution of eosinophils in respiratory diseases.

## Figures and Tables

**Table 1:** Current and potential future biomarkers for severe asthma.

Biomarker	Utility	Limits	Associated Biologic Agents	References
**Sputum eosinophil count**	Indicator of: - airway eosinophilia - type 2 inflammation Predictive of: - exacerbations - response to biologics targeting IL-5	- difficult to collect - requires patient collaboration - needs specialized equipment	Omalizumab Mepolizumab Reslizumab Benralizumab	[50,51]
**Blood eosinophil count**	Indicator of: - airway eosinophilia, remodeling, and hyper-responsiveness Predictive of: - corticosteroids and biologics targeting IL-5 outcome - correlate with the risk of exacerbation	- low specificity - several confounders (e.g., sex, race/ethnicity, smoking, comorbidities) - needs several measurements - requires specialized laboratory	Mepolizumab Reslizumab Benralizumab	[52,53]
**FeNO**	Indicator of: - poor compliance Predictive of: - corticosteroids - biologics targeting IL-13 outcome - exacerbations	- several confounders (e.g., age, smoking, height sex respiratory infections)	Dupilumab Tepezelumab	[54,55]
**Periostin**	Indicator of: - T2 inflammation - airway remodeling Predictive of: - potential prognostic factor	- low specificity	Omalizumab Lebrikizumab Tralokinumab	[56,57]
**MicroRNAsg ^a^**	Predictive of: - asthma severity - risk of exacerbation - asthma endotype (T2-high vs. T2-low) Possible therapeutic target	- not available in clinical practice - not established specificity - specialized equipment needed	NA	[58,59]
**Mast cell count/** **Tryptase**	Indicator of: - asthma endotype Predictive of: - response to omalizumab - severity of the disease	- not validated - specialized equipment needed	Omalizumab	[60]
**IL-13**	Predictive of: - response to anti-IL-13 biologics	- levels are affected by common medication - standardization of sampling and analysis needed - requires specialized laboratory	Lebrikizumab Tralokinumab	[60,61,62]
**INFγ/IL-6 gene** **signatures**	Predictive of: - novel phenotypes (IL6 high and IFNγ- high phenotype) - exacerbations - peripheral and airway eosinophilia - increased immune cell infiltration of the airway submucosa	- difficulty and risk of collecting samples of tissue during bronchoscopy - not established specificity	NA	[63,64]
**VOCs**	Predictive of: - exacerbations - asthma phenotype - response to steroid therapy.	- collection needs standardization and analysis validation - influenced by exogenous factors (e.g., food, bacteria, environmental contaminants)	NA	[65,66,67]

^a^ miR-155, miR-146a, miR-21, miR-1248, and miR-210; FeNO: fractional exhaled nitric oxide; IL: interleukin; miRNA: microRNA; NA: not applicable; VOC: volatile organic compounds.

**Table 2:** Current and future biological therapies for type 2-high severe asthma.

Biological Therapy	Target	Administration Route	Currently Approved for SA	Other Indications	References
Omalizumab	IgE	sc	YES	CRwNP, CSU	[156,159,160]
Mepolizumab	IL-5	sc	YES	EGPA, CRwNP	[156,161]
Reslizumab	IL-5	iv	YES	None	[162]
Benralizumab	IL-5Rα	sc	YES	None	[163]
Dupilumab	IL-4Rα	sc	YES	AD, CRwNP	[100]
Tezepelumab	TSLP	sc	YES	None	[164]
Itepekimab	IL-33	sc	NO (Phase 2 trial)	NA	[165,166,167]

AD: atopic dermatitis; CRwNP: chronic rhinosinusitis with nasal polyps; CSU: chronic spontaneous urticaria; EGPA: eosinophilic granulomatosis with polyangiitis; sc: subcutaneous injection; iv: intravenous injection; TSLP: thymic stromal lymphopoietin.

## Data Availability

No new data were generated or analyzed for this review article.
